# Volume and effectiveness assessment of articain 4% versus mepivacaine 2% used in third molar surgery: randomized, double-blind, split-mouth controlled clinical trial

**DOI:** 10.4317/medoral.23780

**Published:** 2020-07-23

**Authors:** Paula Carolina de Almeida, Fernando Vagner Raldi, Fábio Ricardo Loureiro Sato, Rodrigo Dias Nascimento, Michelle Bianchi de Moraes

**Affiliations:** 1 Master student of the Master's Program in Science and Technology applied to dentistry of the Faculty of Dentistry, São Paulo State University (Unesp), São José dos Campos Campus, SP, Brazil; 2 PhD. Professor in Department of Diagnosis and Surgery of the Faculty of Dentistry, São Paulo State University (Unesp), São José dos Campos Campus, SP, Brazil

## Abstract

**Background:**

The different indications for extraction of the lower third molars, require resources to manage pain and discomfort, such as, for example, adequate anesthetic techniques, and the type of anesthetic used can influence the management of pain in tooth extractions. Few studies in the literature compare the anesthetics 4% articaine hydrochloride and 2% mepivacaine hydrochloride showing evidence that both allow for successful pain management. This study sought to compare the volume, efficacy and safety of these two anesthetic drugs, both associated with epinephrine at a ratio of 1:100,000, used in the extraction of lower third molars.

**Material and Methods:**

A controlled, clinical, split-mouth compared these both local anesthetics in a sample of 20 patients requiring bilateral extraction of teeth. Pain was the main parameter to be assessed by means of the visual analogue scale (VAS) applied during and immediately after the surgery. Hemodynamic parameters, adverse events, presence of paresthesia and satisfaction of patients and surgeon were also analysed.

**Results:**

Pain management was more effective with mepivacaine up to two hours after surgery (*p*=0.014), whereas the surgeon was more satisfied with the use of articaine during divulsion and suture (*p*<0.05). However no statistically significant differences were found between both anesthetics regarding pain perception.

**Conclusions:**

It was observed that both anesthetics are efficient and safe in the management of pain for extraction of third molars, in which less amount of mepivacaine is needed. The satisfaction of patients and surgeon was the same for both anesthetics, with articaine being highlighted during divulsion and suture.

** Key words:**Pain, third molar, local anaesthesia, paresthesia.

## Introduction

In the literature there are different indications for extraction of lower third molars, ranging from presence of local infectious processes to prevention against emergence of lesions in the region (1,2,3). For tooth extraction, it is indispensable to use resources to manage pain and discomfort such as adequate anaesthetic techniques (4,5). Duration and extension of the extraction procedure, patient’s systemic health conditions and type of anaesthetic used are factors which can influence the management of pain in tooth extractions (5,6,7).

There are a variety of anaesthetics which can meet the specific requirements of different clinical procedures, among them 4% articaine chloridrate and 2% mepivacaine chloridrate, both in association with epinephrine at a ratio of 1:100,000. These are anaesthetic salts of amide group, which are largely used in the dental practice and whose clinical safety has already been tested and proved elsewhere (8-12).

There are a few studies in the literature comparing these anesthetic drugs and showing evidence that both allow a successful pain management (11,13,14).When comparing the volume of drug used and the anaesthetic action, studies have shown that there is a correlation between more use of drugs and greater anaesthetic efficacy in the pain management (15). Authors who evaluated changes in hemodynamic parameters using anesthetics found greater safety when lower volume and concentration of vasoconstrictor were employed (16).

Despite their clinical success and availability, tooth extractions can cause morbidity and discomfort to the patient (17). In addition, changes in haemodynamic parameters may occur (18,19) and therefore it is paramount to carefully conduct a local anaesthetic procedure for surgery in order to minimise adverse events.

Evaluation of and comparison between 4% articaine and 2% mepivacaine can help both patients and surgeons by assisting in the selection of anaesthetic drugs so that discomfort can be minimised and adverse events better managed during and after surgery, which consequently benefits the parties involved (9,11,13).

The objective was to compare the volume used of these two anesthetic drugs in relation to their effectiveness on pain intensity during and after surgery, hemodynamic parameters and patient and surgeon satisfaction with the anesthesia generated, as well as possible adverse effects.

## Material and Methods

This randomised, controlled, double-blind, clinical, split-mouth study was submitted to the ClinicalTrials.gov according to protocol number NCT 03384160 and approved by the local research ethics committee according to protocol number CAAE 80943517.8.0000.0077. The methodology used followed the CONSORT statement guidelines (20,21). Informed consent form was signed by each patient after explanation of the nature, risks and benefits of the clinical investigation.

The sample population consisted of patients attending the Oral and Maxillofacial Surgery Clinic of the Institute of Science and Technology of State University of São Paulo (ICT - UNESP) for bilateral extraction of included or semi-included lower third molars, as described by Pell & Gregory [1933] and Winter [1926] in theirs Classification (22-24).

- Inclusion Criteria

1. Systemically healthy patients aged between 16 and 40 years old;

2. Patients with no morphological or pathological change in the oral cavity;

3. Periodontally healthy patients;

4. Patients needing bilateral extraction of included or semi-included lower third molars, as described by Pell & Gregory [1933] and Winter [1926].

- Exclusion Criteria

1. Patients with systemic health problems (e.g. cardiovascular changes, blood dyscrasias, immunodeficiency, diabetes, etc.) contra-indicating the surgical procedure;

2. Patients taking medications which can interfere with wound healing, pain perception and anaesthetic use or contra-indicate surgical procedure;

3. Pregnant or breastfeeding patients;

4. Patients with oral lesions, opportunistic infections or using topical medications on the oral region;

5. Patients who are smokers;

6. Patients with history of allergic reactions (i.e. hyper-sensibility) to anaesthetics;

7. Patients allergic to medications used in the post-operative protocol.

Screening was performed within a 6-month period. Initially, 98 patients were selected. After detailed anamnesis and panoramic radiographic evaluation, 76 patients were excluded and 22 were considered eligible for study. However, two cases of dropouts occurred during the study, in which one was not justified and the other was due to pregnancy. The final number of patients was 20, which thus involved 40 toot extractions.

The sample size was calculated as described on the site www.sealedenvelo.com, in which a minimum amount of 34 teeth extracted from 17 patients was the number needed to detect a study power of 90% at a significance level of 5% (11,25).

- Group Distribution

The eligible patients were randomly distributed into two groups by using the Microsoft Excel 2007 software. The right side of all patients was chosen for the initial surgery (i.e. extraction of tooth #48) and the left side was chosen for the second surgery (i.e. extraction of tooth #38). Local anaesthesia was performed with an anaesthetic drug different to that used in the opposite side (i.e. on either right or left side) so that each patient had one of the sides in the different groups of anaesthetics. Data on the respective distributions were placed in opaque envelopes and sealed. Treatment and type of anaesthetic administered in each group were the following:

Group 1 - Patients undergoing extraction of the lower third molar under local anesthesia with 4% articaine chloridrate and epinephrine at a ratio of 1:100,000.

Group 2- Patients undergoing extraction of the lower third molar with local anesthesia with 2% mepivacaine chloridrate and epinephrine at a ratio of 1:100,000.

- Surgical Procedure

Prior to the surgical procedure, 15 ml of chlorhexidine digluconate 0.12% solution was used for intra-oral disinfection and chlorhexidine digluconate 0.2% solution for peri-oral disinfection of the skin. Next, a sterile operative field was used to define the contamination area.

A single experienced surgeon (FVR) was assigned to perform the tooth extractions. Local anaesthesia was administered by using the inferior alveolar nerve block technique, which blocks the lingual and buccal nerves. Anaesthetic action was determined and recorded when the patient reported loss of sensibility in the lower lip and in the anterior half of the tongueon the side wherethe anaesthetic was administered. Patients and surgeon had no knowledge on the type of anaesthetic drug being administered as another researcher placed the anaesthetic cartridges into de syringes, according to previous random selection.

The amount of anaesthetic drug was standardised in two cartridges (3.6 ml) for each side. In those cases in which anaesthesia was complemented, the amount of additional anaesthetic drug and form of administration were recorded on the data collection sheet.

The procedure was initially performed by the surgeon, who made a straight incision 1.0 cm distal from the second lower molar towards the central sulcus, followed by intra-sulcular incision to the inter-dental papilla between second and first molars made with a #15 blade.

Complete divulsion of the envelope flap was performed by using a Molt elevator in order to provide an adequate surgical field for the extraction procedure. After divulsion, osteotomy was made to expose the tooth until crossing the prosthetic equator by using a #4 spherical carbide bur at high rotation under abundant irrigation with 0.9% sodium chloride solution. Tooth-section was performed when necessary. Dental avulsion was performed by using straight Seldin and Potts elevators, followed by inspection of the alveolus and abundant irrigation with 0.9% sodium chloride solution. The procedures were completed by suturing the repositioned flap with a 3.0 silk suture (Ethicon, Johnson & Johnson, São José dos Campos, SP, Brazil).

All the patients followed only one post-operative medication protocol, which was initiated two hours after the surgery procedure, namely:Amoxicillin 500 mg, one Tablet every 8 hours for 7 days; Sodium diclofenaco 50 mg, one Tablet every 12 hours for 5 days;Paracetamol 750 mg, one Tablet every 6 hours for 3 days.

The surgeries on each side of the patient’s face were performed at a minimum interval of 20 days.

- Data Collection

Only one evaluator was appointed to collect and record data on the patients before, during and after the surgeries. The same evaluator administered the questionnaire to the surgeon to record his/her satisfaction withthe surgery and anaesthesia, including anaesthetic volume for initial and complement nerve blockade in each procedure and time elapsed before anaesthetic action.

- Patient-Centred Parameters

Immediately before the initial anaesthetic action, the patient’s haemodynamic parameters were measured as follows: blood pressure, heart rate and oximetry by using a digital device (OmronCorp. Osaka, Japan). These procedures were repeated during and after the surgeries at 5, 20 and 70 minutes after administration of the anaesthetic drugs.

Soon after administration of the anaesthetic solution, the patient was observed for possible adverse reactions such as dizziness, shakes, pains, shivers, agitation, depression, allergic reactions or any other event requiring intervention. The reactions observed were immediately recorded.

The subjective evaluation of pain was recorded by using the visual analogue scale (VAS). The patient was instructed on how to answer the scale, in which a vertical line indicated numbers ranging from 0 to 10. The subjective evaluation of pain was performed at two periods, namely, during and immediately after the surgery (i.e. at 0, 2, 4 and 6 hours) by means of a questionnaire containing two Likert-type questions with five answer options, the patient’s satisfaction with anaesthesia and surgery was recorded. The patient was instructed to mark only one answer among the five options as follows: fully unsatisfied, partially unsatisfied, indifferent, partially satisfied and fully satisfied. Each option corresponded to 1, 2, 3, 4 and 5 points, respectively. Lastly, this same questionnaire asked the patient to indicate the time when he or she perceived loss of anaesthetic sensibility and the time when he or she experienced the first sensation of pain following the surgery, thus allowing us to calculate the duration of anaesthesia.

- Surgeon-Centred Parameters

Soon after the surgery, the surgeon was given a form containing two Likert-type questions (i.e. from 1 to 5 points), one aimed at recording the surgeon’s subjective evaluation and degree of bleeding during surgery and other aimed at recording the surgeon’s satisfaction with anaesthesia. In addition, the form contained a checklist with each surgical procedure step for attribution of grades and further two Likert-type questions on satisfaction (i.e. from 1 to 5 points) as follows: fully unsatisfied, partially unsatisfied, indifferent, partially satisfied and fully satisfied. Each option corresponded to 1, 2, 3, 4 and 5 points, respectively. In this way, the higher the degree of satisfaction the higher the score, as described in the Likert scale (26).

- Statistical Analysis

Shapiro-Wilk’s test was used to assess whether the resulting data followed a Gaussian distribution before being submitted to statistical analysis with IBM SPSS Data Editor software, version 11 (IBM Corp., Armonk, USA). Student t-test was applied to each variable being analysed and chi-square to nominal variables. All statistical tests were performed at a significance level of 5%.

## Results

- Patient Recruitment

Patients aged 16-40 years old participated in the study, being 17 females (85%) and three males (15%). All of them took part in the Groups 1 and 2 simultaneously.

The surgical extraction of the lower third molars on the right side (i.e. tooth #48) was performed nine times under anaesthesia with articaine and 11 times under anaesthesia with mepivacaine, whereas the one on the left side (i.e. tooth #38) was performed 11 times with articaine and nine times with mepivacaine.

- Amount of Anaesthetic Solution and Mean Times

The mean amount of anaesthetic solution used was slightly greater in Group 1, with a difference of 0.34 ml, but which was not statistically significant. The mean times for length of surgery, anaesthetic on set time and duration of anaesthesia were not statistically different between the groups, although Group 1 had a more rapid anaesthetic onset (Table 1).

**Table 1:**
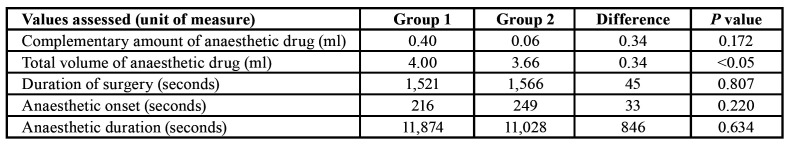
Assessment of anaesthetic efficacy in terms of amount and experimental periods.

- Pain Assessment

The comparison of pain perception during and immediately after (at 0, 2, 4and 6 hours) surgery between both groups was the main variable of the study. The greater difference between both groups was found during the surgery, in which Group 1 had a mean value of 1.10 point compared to 2.05 points of Group 2, thus opposing the values found immediately after the surgery in which pain management was greater in Group 2. However, these differences between both groups were not statistically significant. A comparative analysis was also performed, showing statistically significant difference immediately after the surgery between 0 and 2 hours (*P*<0.05) in both groups. One can notice that pain was more intensively experienced between 2 and 4 hours after the surgery in both groups, but with no statistical difference (Table 2).

- Blood Pressure, Heart Rate and Oximetry

With regard to blood pressure, variations were found at the different experimental times despite the lack of statistically significant differences between both groups or between these periods. However, Group 1 showed a tendency towards greater variation in diastolic blood pressure immediately before the surgery and 5 minutes after as well as between 20 and 70 minutes after. Despite the variation found in the parameters heart rate and oximetry between the groups, there were no statistically significant differences. We have also analysed the heart beat variations and found that there were greater variations between the experimental times immediately before the surgery and 5 minutes after application of anaesthesia in both groups, with statistically significant difference in Group 1 between these periods. On the other hand, the variations in heart rate between 5 and 20 minutes following anaesthesia were statistically significant in both groups (Table 3).

**Table 2:**
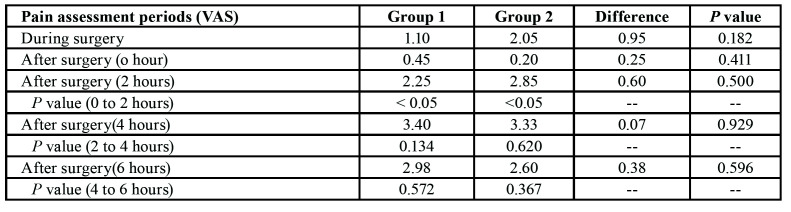
Assessment of pain perception (VAS) during and immediately after the surgery.

**Table 3:**
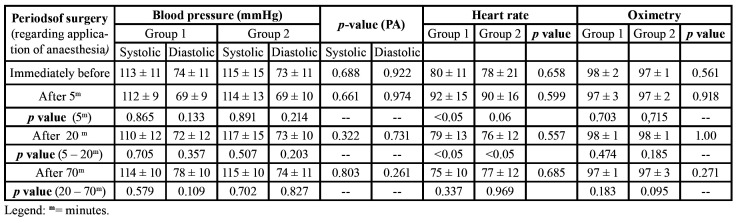
Assessment of haemodynamic parameters: blood pressure (BP), hear rate and oximetry.

- Adverse Events

The following manifestations of adverse events were recorded during surgery, namely: pain (Group 1, n = 12 and Group 2, n = 9), pressure sensation (Group 1, n = 1 and Group 2, n = 0), tremor (Group 1, n = 0 and Group 2, n =1) and excessive bleeding (Group 1, n = 0 and Group 2, n = 2). No statistically significant differences were found between both groups regarding frequency of pain (*p*=0.749). Bleeding during surgery was assessed by the surgeon according to a Likert scale (Jamieson, 2004) and the difference between both groups (Group 1 = 1.42 and Group 2 = 1.65) was not statistically significant (*p*=0.283).

Occurrence of paresthesia was observed in the post-operative controls, with three cases in Group 1 and one case in Group 2. Statistical analysis showed no significant difference between both groups (*p*=0.292).

These cases of paresthesia were reversible within a 30-day period, in which no systemic medication was used and a low-potency laser (Photon Laser III, DMC, São Carlos, São Paulo, Brazil) operating at 3 J/cm2was applied onto the affected area every 7 days in four sessions, on average.

- Satisfaction of Patients and Surgeon

Analysis of the degree of the patient’s satisfaction showed that Group 1 had 4.09 points for anaesthesia and 5 points for surgery, whereas Group 2 had 4.75 and 5 points, respectively. With regard to the patient’s satisfaction with the anaesthetic used, no statistically significant differences (*p*=0.223) were found between the two groups.

With regard to the surgeon’s satisfaction with the anaesthetic used and surgery, the mean values in Group 1 were 4.85 points for the former and 5 points for the latte. In Group 2, the mean values for anaesthetic used and surgery were, respectively, 4.70 and 4.75 points. In the comparison between both groups, no statistically significant differences were found for anaesthetic (*p*=0.268) and surgery (*p*=0.503). The degree of satisfaction of the surgeon during each period of surgery was assessed, and only the variables divulsion and suture had statistically significant differences. There was a tendency towards greater satisfaction with the surgeries performed in Group 1 (Table 4).

**Table 4:**
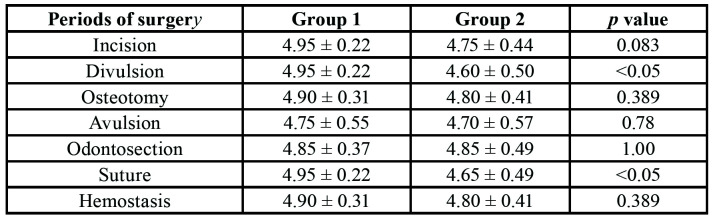
Assessment of the surgeon’s satisfaction with the periods of surgery.

## Discussion

The present study made standardisation oftooth extraction technique and surgery team, including patient selection by bilateral similarity, in order to decrease differences from individual variations and consequently allow a reliable comparison of the evaluated parameters (11,15).

The main parameter of study was the comparison of pain perception, which was measured with VAS. It was possible to visualise a variation between Group 1 (1.10 point) and Group 2 (2.05 points), in which the greatest difference was observed during the surgery despite the lack of statistical significance. Another study also found differences between articaine and mepivacaine regarding pain perception, but no statistically significant difference was observed during and fter surgery (11). We have analysed pain perception at these different periods and found statistically significant differences between 0 and 2 hours after the surgery (*p*<0.05) for both anaesthetic drugs. In fact, there was a tendency towards an increase in the rate of pain perception between 2 and 4 hours after surgery for both anaesthetic drugs. Therefore, it was possible to observe that pain management was more effective within 2 hours after the surgery, which corresponds to the period of better pain management as reported by other study, where all patients had pain scores less than 10 points within the first post-operative hour, regardless of the local anaesthetic drug used (11). These values are close to those reported by our patients immediately after the surgery (i.e. 0 hour), which were 0.45 and 0.20 points in Group 1 and Group 2, respectively.

With regard to the anaesthetic onset time, there is a difference between articaine and mepivacaine in which 3.36 minutes and 4.09 minutes were observed, respectively. Moreover, anaesthetic onset times of 2,98 minutes for articaine and 4,22 minutes for mepivacaine were reported elsewhere (13), which differ from the results found by a clinical study showing that both drugs had a shorter onset time of 2.30 minutes (11).

Duration of anaesthesia is another important parameter for assessment of the pain management, with the present study finding mean times of 3 hours, 17 minutes and 54 seconds for articaine and 3 hours, 3 minutes and 48 seconds for mepivacaine. A study also showed that articaine had a longer duration of action than mepivacaine (11). Another study reported a longer duration of action for 2% mepivacaine compared to articaine, but these results are difficult to compare because they involved healthy soft tissues with no surgical intervention (27).

Some adverse events were observed during the surgeries, but with no statistically significant differences between the anaesthetic drugs (*p*= 0.749), although other clinical studies reported no adverse reactions during surgical procedures (11,25). Due to these adverse events, it was necessary to complement the anaesthesia during surgery in both groups by using, on average, 0.40 ml of articaine and 0.23 ml of mepivacaine. Considering the initial amount of anaesthetic drugs used, there was a total volume of 4.00 ml in Group 1 compared to 3.66 ml in Group 2. In this way, one can observe that a smaller dose of anaesthetic was needed for mepivacaine (*p*<0.05). However, a clinical study usedlower volumes of anaesthetic drugs in a comparison between articaine and mepivacaine, in which 2.7 ml was determined for both solutions, and in only one case anaesthesia was complemented with 0.9 ml of mepivacaine for pain management during surgery (11).

Monitoring of haemodynamic parameters has allowed us to observe variations in vital signs common to the use of anaesthetics (11,28). We have observed a greater tendency towards variation in diastolic blood pressure in Group 1, but variations between both groups as well as between experimental times were not statistically significant. These results are corroborated by findings of transient increases and decreases in vital signs, which were not clinically significant between each other and between the treatment groups (11,28). In the present study, however, we have observed greater variations in heart rate immediately before and 5 minutes after application of anaesthesia in both groups, with a statistically significant difference being found in Group 1. Variations between 5 and 20 minutes were statistically significant in both groups of anaesthetic drugs.

With regard to the occurrence of paresthesia, Group 1 had three cases and Group 2 had one case, but with no statistically significant difference between both groups (*p*=0.292). Nevertheless, this raised discussion on the association between the use of 4% articaine and occurrence of paresthesia as there are studies reporting that cases of paresthesia associated with local anaesthesia are extremely rare if one considers the entire amount of anaesthetic drugs used in surgeries worldwide. However, this is opposed to data from prospective studies with limited sample size or retrospectively collected as they may be incomplete and potentially reflect in bias of notification, thus meaning that there are data suggesting apossible cause-and-effect relationship rather than a definitive evidence of it (29). This discussion is enhanced when there are reports of paresthesia related to local anaesthesia, as those recorded in the FDA Adverse Event Reporting System between 2004 and 2011. Due to the significant relationship between paresthesia and local anaesthetics like 4% articaine and 4% prilocaine, including signs of disproportionality, a study proposed that more specific research is needed to confirm or reject the cause-and-effect relationship between articaine solutions and paresthesia (30).

When the degree of the patients’ and surgeon’s satisfaction with anaesthesia used and surgery itself was assessed, no statistically significant differences were found between both anaesthetic drugs. The satisfaction of the surgeon was assessed for each period of surgery, in which statistically significant differences were found for the variables divulsion and suture. However, one can note a tendency towards greater satisfaction with surgeries performed in Group 1, which does not devaluate the use and amounts of mepivacaine either.

With this study, it was possible to observe that articaine and mepivacaine were similarly effective and safe, in addition to providing pain management during extraction of lower third molars, with the latter anaesthetic drug requiring less total volume. With regard to the patients’ and surgeon’s satisfaction, both anaesthetics were considered satisfactory, although articaine was highlighted by the surgeon during divulsion and suture.
